# High prevalence of *Trypanosoma cruzi* infection in shelter dogs from southern Louisiana, USA

**DOI:** 10.1186/s13071-019-3572-y

**Published:** 2019-06-25

**Authors:** Ardem Elmayan, Weihong Tu, Brandy Duhon, Preston Marx, Wendy Wolfson, Gary Balsamo, Claudia Herrera, Eric Dumonteil

**Affiliations:** 1 Department of Tropical Medicine, School of Public Health and Tropical Medicine, Tulane University, New Orleans, Louisiana USA; 2 Vector-Borne and Infectious Disease Research Center, Tulane University, New Orleans, Louisiana USA; 3 School of Veterinary Medicine, Louisiana State University, Baton Rouge, Louisiana USA; 4 Division of Microbiology, Tulane National Primate Research Center, Tulane University, Covington, Louisiana USA; 5 Infectious Disease Epidemiology Section, Office of Public Health, Department of Health, New Orleans, Louisiana USA

**Keywords:** Chagas disease, American trypanosomiasis, Canine, Heart disease, Parasite

## Abstract

**Background:**

Chagas disease is a zoonotic disease caused by the protozoan parasite *Trypanosoma cruzi*. The role of dogs as sentinels has been proposed in multiple regions, as they are a domestic reservoir for *T. cruzi.* Our objective was to determine the prevalence of *T. cruzi* infection in shelter dogs from southern Louisiana, and assess its magnitude and distribution.

**Results:**

A total of 540 dogs were enrolled, from 20 animal shelters, and tested for *T. cruzi* infection by serological tests (rapid test, ELISA and western blot) and PCR. We documented a high prevalence of *T. cruzi* infection with at least 6.9% (95% CI: 5.0–9.3%) seropositive and 15.7% (95% CI: 12.9–19.1%) PCR-positive dogs. Serological tests showed limited agreement, and concordance between serology and PCR was higher when considering reactivity to single serological tests. *Trypanosoma cruzi* infection was distributed evenly among shelters. Infection was significantly correlated with age (*R*^2^ = 0.99), indicating an incidence of new cases of 2.27 ± 0.25% per year.

**Conclusion:**

*Trypanosoma cruzi* infection is a significant and widespread veterinary problem in shelter dogs in the region, although it is mostly unnoticed by health professionals. This highlights the need for greater awareness of *T. cruzi* infection among the veterinary community and dog owners.

**Electronic supplementary material:**

The online version of this article (10.1186/s13071-019-3572-y) contains supplementary material, which is available to authorized users.

## Background

Chagas disease is a zoonotic disease caused by the protozoan parasite *Trypanosoma cruzi*. It is transmitted to mammalian hosts through the feces of infected triatomine bugs during blood-feeding. It is a major public health problem in the Americas, with over 6 million cases in Latin America [1]. It is also of growing concern in the USA, where there are over 300,000 cases, and more active surveillance is leading to the identification of an increasing number of locally acquired infections [2, 3]. Human spillover infections derived from zoonotic transmission cycles may thus be occurring more frequently than currently acknowledged and improved surveillance should help define the risk for parasite transmission to humans. In particular, the role of dogs as sentinels for human infection has been proposed in the USA as well as in multiple settings in Latin America, since dogs represent one of the main domestic reservoir for *T. cruzi* parasites [4–6].

*Trypanosoma cruzi* infection in dogs has been well documented in Texas, since at least the 1980s [7, 8] and domestic transmission cycles have been identified [9]. Multiple seroprevalence studies have evidenced a significant level of infection in different canine populations, ranging from 7.4 to 18.2%, up to 57.6% in some kennels [10–16]. Triatomine blood meal analysis also documented that bugs frequently feed on canines in kenels [17, 18].

Nonetheless, in spite of the extensive distribution of triatomine vectors in the southern half of the USA and a wide distribution of zoonotic *T. cruzi* infection in a wide range of mammalian species, only a limited number of studies have been conducted outside of Texas [7, 15, 19–22]. The first canine case in Louisiana was reported in 1980 [23], and a few subsequent studies reported a seroprevalence of 1.1% in domestic dogs in New Orleans [24], 2.3% in dogs from animal shelters and 4.7% in rural dogs [25], and 12–62% in some kennels [26], making it difficult to extrapolate such data. Occasional cases of canine *T. cruzi* infection have also been reported in other states such as Oklahoma [27] and Virginia [28–30]. Thus, the current magnitude of canine infection with *T. cruzi* in the USA is difficult to establish, in spite of the multiple reports indicating that infection is present [15].

Our objective was to determine the prevalence of *T. cruzi* infection in shelter dogs from southern Louisiana, and assess the magnitude and distribution of the infection. Such information is key for veterinarians to improve disease surveillance and diagnostics, and for providing adequate veterinary care to infected dogs. It is also of importance for an improved surveillance of human disease as well, given the role of dogs as *T. cruzi* reservoirs.

## Methods

### Participating shelters and sample collection

A convenience sample of 20 animal shelters participating in the Louisiana State University (LSU) shelter programme were included in the study. The Shelter Medicine programme provides veterinary services to local animal shelters and rescue groups, which include spay/neuter surgeries, physical exams and expertise on infectious disease outbreaks. Participating shelters covered most of the southern part of Louisiana, with shelters in Acadia, Ascension, Calcasieu, East Baton Rouge, Iberia, Iberville, Jackson, Lafourche, Livingston, Natchitoches, Orleans, St. Landry, St. Martin and Tangipahoa parishes. We used excess blood samples in citrate tubes collected during the routine veterinary care of the dogs and aliquots were stored at 4 °C until processed for analysis. A total of 540 dogs were enrolled in the study, ranging from 5 to 49 per shelter, by convenience.

### Blood samples processing and analysis

Upon arrival of blood samples to the laboratory, an aliquot was mixed an equal volume of 6 M guanidine HCL and stored at room temperature. We also used 10 µl of whole blood for testing *T. cruzi* infection using Stat-Pak immunochromatic rapid test [26, 31] as instructed by the manufacturer (Chembio, Medford, NY, USA). Plasma was prepared from the remaining blood for additional serological testing by ELISA and Western blot.

### ELISA

ELISA tests were run as previously described [32] using whole parasite lysate from a local strain (WB1) as antigen. Briefly, ninety-six well microplates were coated overnight at 4 °C with 10 µg/well of *T. cruzi* parasite lysate in carbonate buffer, washed three times with PBS, and blocked with 1% BSA and 0.05% Tween 20 in PBS for 1 h at 37 °C. After three additional washes, a 1:500 dog serum dilution was added in duplicate wells and incubated for 1 h at 37 °C. Wells were then washed 3 times, and incubated with a peroxidase-labeled rabbit antibody against dog IgG (Sigma-Aldrich, St. Louis, MO, USA) at a 1:5000 dilution, for 30 min at 37 °C. After a three final washes, 3,3′,5,5′-tetramethylbenzidine substrate in DMSO and phosphate-citrate buffer (pH 5.0) with 30% hydrogen peroxide were added and incubated for 30 min at room temperature in the dark. Reactions were stopped with 2 M H_2_SO_4_, and plates were read at 450 nm in an ELISA plate reader.

### Western blot

Cultured *T. cruzi* parasites were lysed in PBS buffer by freeze-thaw cycles. After clearing debris by centrifuging at 14,000×*g* at 4 °C, protein concentration of the extracts was determined by spectrophotometry (NanoDrop 2000, Thermo Fisher Scientific, Waltham, MA, USA). The parasite lysate was denatured with SDS sample buffer and separated in 12% SDS-PAGE. Proteins were transferred onto nitrocellulose membranes using a Bio-Rad mini protein-II wet transfer unit. The transferred membranes were incubated with the blocking solution (5% nonfat dried milk dissolved in PBS-T buffer) for 1 h at room temperature then incubated with dog blood serum (1:200 dilution, in blocking buffer) overnight at 4 °C with gentle agitation. Membranes were washed three times with PBS-T buffer, then incubated with the secondary antibody anti-dog IgG (whole molecule)-Peroxidase produced in rabbit (1:5000 dilution, Sigma-Aldrich, St. Louis, MO, USA) for 1 h and washed four times. Signal detection was performed with an enhanced chemiluminescence kit (Clarity Western ECL Substrate kit, Bio-Rad, Hercules, CA, USA). Images were captured using Image Quant LAS 4000, with exposure times of 2 min.

### DNA extraction and PCR diagnostic

DNA was extracted from 0.2 ml of blood-guanidine samples using Qiagen DNAeasy Extraction kit (Qiagen, Germantown, MD, USA) according to the instructions of the manufacturer. The presence of *T. cruzi* DNA was assessed by PCR targeting kinetoplast DNA as described before [33, 34].

### Data analysis

We calculated the proportion of reactive samples for each of the serological and molecular tests. Proportion data are presented as percentages ± 95% confidence interval (CI). Agreement between tests was assessed by Kappa index. *Trypanosoma cruzi* seropositivity was defined as confirmed for dogs with at least 2 reactive serological tests. Continuous variables such as dog age are presented as the mean ± standard error of the mean (SEM), and compared between groups using Student’s t-test. Changes in seroprevalence with age were fitted by semilog regression and the goodness-of-fit was assessed by *R*^2^. The average increase in seroprevalence per year was used to estimate incidence. A map of the distribution of seropositive dogs was elaborated in QGIS 3.4, and EPA ecoregions were used (https://www.epa.gov/eco-research/ecoregion-download-files-state-region-6) to assess potential associations between seroprevalence and ecological characteristics surrounding the shelters. Comparison of seropositivity among shelters and ecoregions was performed by Chi-square tests.

## Results

### Serological diagnostics of *T. cruzi* infection

We collected a total of 540 blood samples from participating shelter dogs. Thirty two out of 539 (6.3%) were reactive using Stat-Pak immunochromatographic rapid test, and 44/539 (8.2%) by ELISA (Table 1). Agreement between the two tests was poor (Kappa index = 0.096). Thus, we used Western blot for confirmatory testing. Again, agreement between ELISA and Western blot tests was poor (Kappa index = 0.061, Table 1). Overall, there were 121/539 dogs (22.4%, 95% CI: 19.1–26.2%) reactive with any one test, and 37/539 confirmed seropositives with at least 2 reactive tests (6.9%, 95% CI: 5.0–9.3%). Male dogs were significantly more infected than females (9.0%, 95% CI: 6.2–12.9 *vs* 3.9%, 95% CI: 2.0–6.7%, respectively, *χ*^2^ = 5.89, *df* = 1, *P* = 0.015).

**Table 1:** Serological testing for *T. cruzi* antibodies in dogs

	Stat-Pak Pos	Stat-Pak Neg	Total	WB Pos	WB Neg	Total
ELISA Pos	6	38	44	30	14	44
ELISA Neg	26	467	493	72	49	121
Total	32	505	537	102	63	165
Kappa		0.096 ± 0.059			0.061 ± 0.059	

*Abbreviations*: WB, Western blot; Pos, number of positive cases; Neg, number of negative cases

**Fig. 1:**
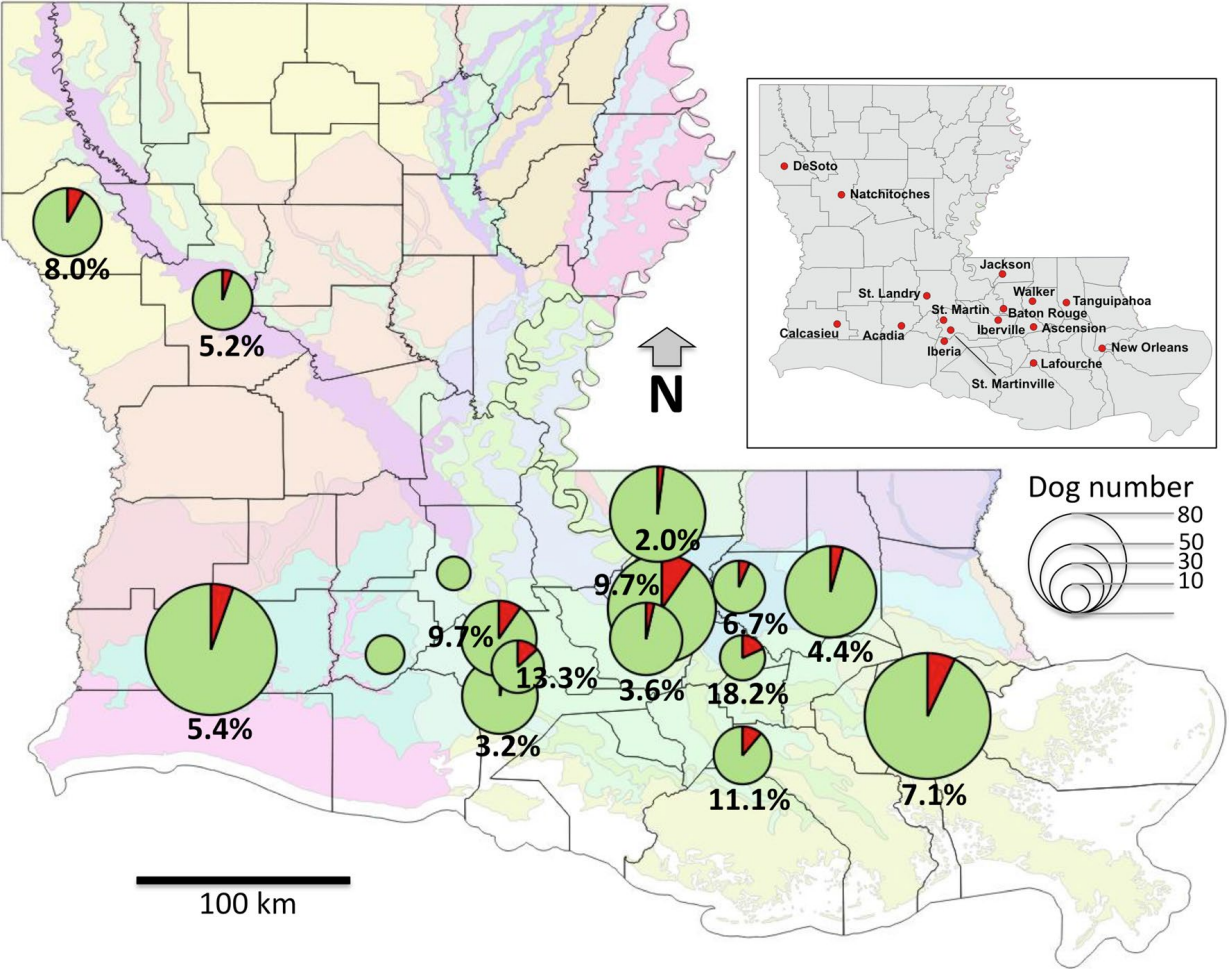
Distribution of *T. cruzi* infection in animal shelters across Louisiana. Insert map: Distribution of Lousiana parishes included in the study. Main map: Distribution of *T. cruzi* seroprevalence in shelter dogs. Pie charts indicate the percentage of seropositive dogs (shown in red) and the size of each chart is proportional to the sample size for the corresponding parish. Background map shows Louisiana parish boundaries and EPA ecoregions (color coded)

The geographical distribution of confirmed seropositive cases varied from no cases in two shelters (in Acadia and St. Landry parishes), up to 18.2% (in Ascension parish), but these differences did not reach statistical significance (*χ*^2^ = 10.375, *df* = 15, *P* = 0.79), indicating that *T. cruzi* infection was evenly distributed across animal shelters from southern Lousiana (Fig. 1). Accordingly, there were no differences in confirmed seropositivity rates according to the ecoregions from southern Louisiana surrounding the shelters (*χ*^2^ = 6.491, *df* = 8, *P* = 0.59).

**Fig. 2:**
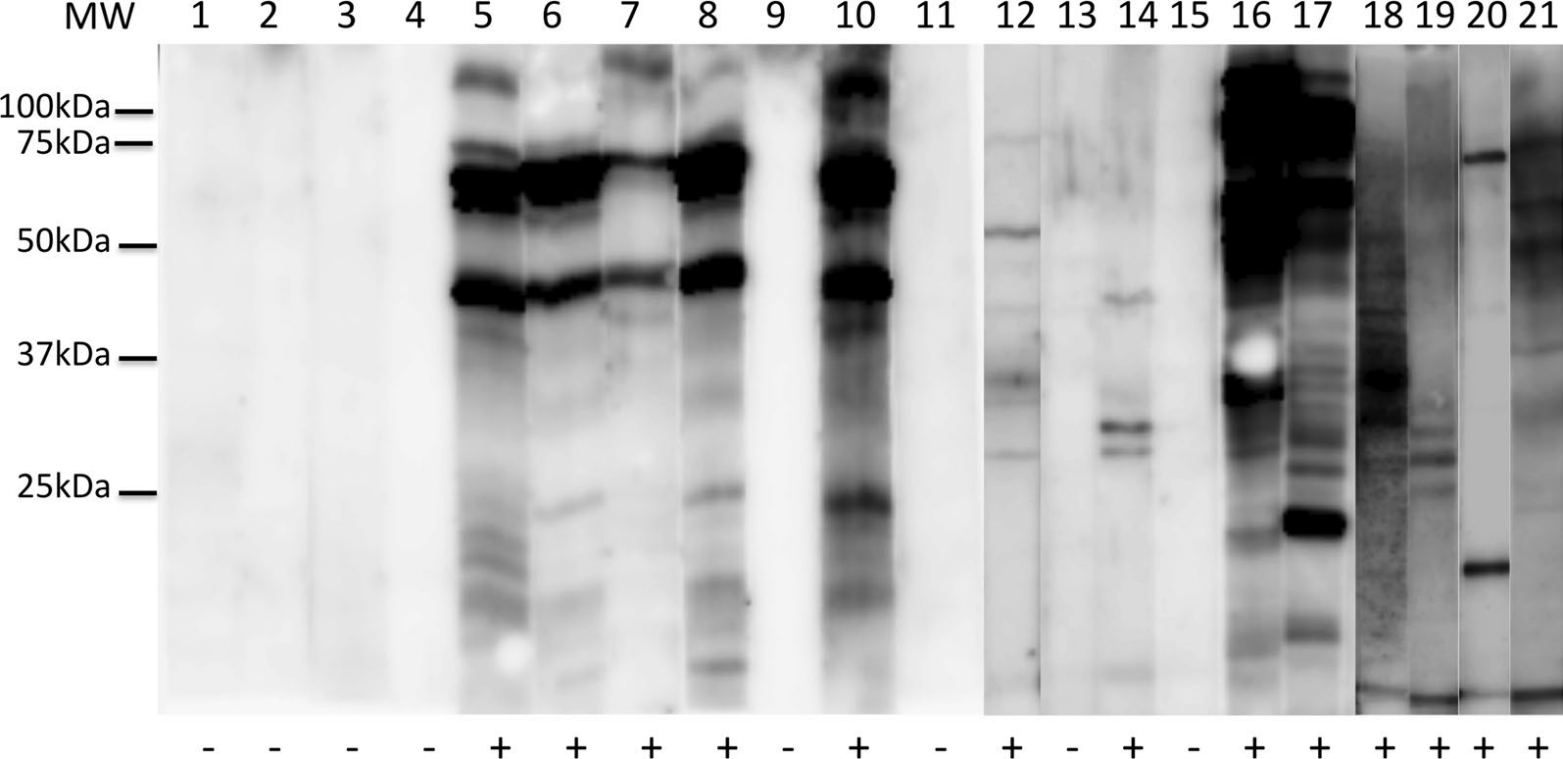
Western blot analysis of antigenic recognition patterns of dog serum. Representative individual dog serums (Lanes 1–21) were tested for *T. cruzi* protein recognition. Positive (+) or negative (−) reactivity is indicated at the bottom of each lane. Note that some dogs showed a very similar band recognition pattern (Lanes 5–8 and 10), but serum from most seropositive dogs recognized widely different parasite antigen patterns (Lanes 12, 14 and 16–21)

Detailed analysis of *T. cruzi* protein bands recognized by dog antibodies in Western blot assays provided some clues to the high discrepancies among serological tests (Fig. 2). Indeed, while serum from a few dogs showed a very similar band recognition pattern (Lanes 5–8 and 10), serum from most seropositive dogs recognized widely different parasite antigens (compare Lanes 12, 14, and 16–21). Also, antigen recognition was focused on very few *T. cruzi* protein bands in several instances (Lanes 14 or 20 for example). This suggested unique interactions between each individual dog and their infecting parasites, leading to widely different antibody profiles and parasite recognition patterns which may not be easily captured by diagnostic tests based on a limited number of parasite antigens.

**Fig. 3:**
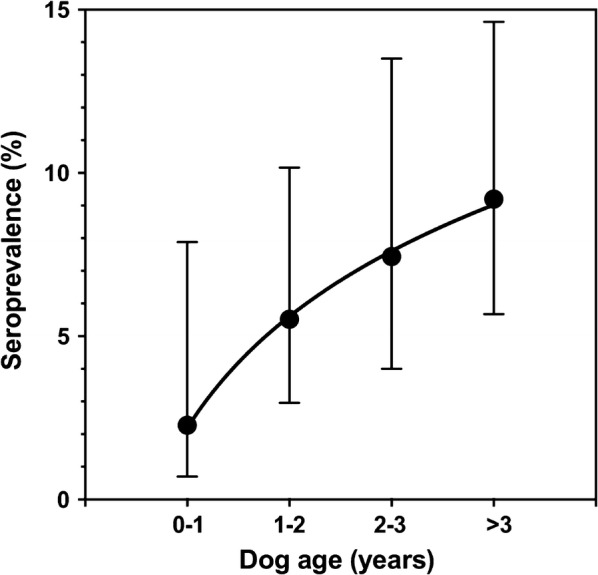
*Trypanosoma cruzi* seropositivity in shelter dogs as a function of age. Seroprevalence is shown as the mean ± 95% CI. Seroprevalence increased with dog age according to: Seroprevalence = 2.19 + 11.35*log(Dog age) (*R*^2^ = 0.99, *P* = 0.001)

Analysis of dog age indicated that confirmed seropositive dogs were significantly older than seronegative dogs (36.0 ± 4.5 *vs* 26.7 ± 0.9 month-old, *t*_(45.6)_ = 1.96, *P* = 0.025). In addition, seroprevalence of infection increased significantly with dog age (Fig. 3, *R*^2^ = 0.99, *P* = 0.001). The average incidence of new infections was of 2.27 ± 0.25 for 100 dogs/year. Discordance among serological tests also seemed to increase with dog age (Kappa index of 0.27, 0.14, -0.06 and 0.13, for dog age 0–1, 1–2, 2–3 and > 3 years-old, respectively).

We further performed PCR detection of *T. cruzi* DNA in dog blood. A total of 85 out of 540 dogs were PCR-positive for *T. cruzi* (15.7%, 95% CI: 12.9–19.1%). This was much higher than detected by serology. Indeed, agreement between PCR and serology was poor (Table 2), as only 6 of the 37 confirmed seropositive dogs were PCR-positive for *T. cruzi*, while 79 seronegative dogs resulted PCR-positive. The agreement between PCR and serology was much higher when we lowered the specificity threshold of the serology by considering the reactivity to a single test instead of two, as up to 41 dogs seroreactive with a single reactive test were also PCR-positive (Table 2), and the Kappa index reached 0.280. This strongly suggested that many of the dogs with a single seroreactive test were not false positives but corresponded to true infections. Agreement of individual serological tests with PCR was low (Additional file 1: Table S1).

**Table 2:** PCR and serological testing for *T. cruzi* in dogs

	Confirmed seropositive (2 tests)	Seronegative	Total	One reactive test only	Seronegative	Total
PCR Pos	6	79	85	41	44	85
PCR Neg	31	424	455	74	381	455
Total	37	503	540	115	425	540
Kappa		0.003 ± 0.039			0.280 ± 0.050	

*Abbreviations*: Pos, number of positive cases; Neg, number of negative cases

## Discussion

We performed here a large scale assessment of *T. cruzi* infection in shelter dogs in Louisiana and observed an average seropositivity of at least 6.9%, based on two reactive tests. This is in agreement with most of previous studies in various canine populations in the southern USA [10–16, 24, 25]. However, we also detected a rather high discordance among tests, comparable to that reported previously in canines in Texas [10]. Variable levels of discordance among tests had been observed previously in dogs in Argentina [31, 35, 36]. Thus, while the Stat-Pak rapid test may be useful for the rapid screening of canines [26, 31], our data suggest that major improvements in serological diagnostic tests are needed for a better surveillance of infection, as noted before for human Chagas disease [37].

The results of our confirmatory Western blot assays may give some clues for this discrepancy. Indeed, the sera from infected dogs recognized widely different parasite protein patterns, indicating very variable profiles of parasite-specific antibodies. This may be due to a combination of dogs’ immune system (DLA) and parasite strains, both of which may be highly variable. So far, most efforts at parasite genotyping in dogs have lead to the detection of TcI and TcIV in the southern USA [10], but a much greater diversity of strains covering TcI, TcII, TcIV, TcV and TcVI has been identified in Louisiana in other mammalian hosts such as rodents [38] and non-human primates [39] when using a more sensitive genotyping method based on next-generation sequencing (NGS) [40]. Infections with such a wide diversity of parasite strains in dogs may in part lead to variable immune responses as we observed. Further attempts at identifying parasite strains infecting dogs in Lousiana should help clarify this point.

The detection of *T. cruzi* DNA by PCR also resulted useful to complement serology, and we detected a high prevalence of PCR positive dogs (15.7%, 95% CI: 12.9–19.1%) due to a highly sensitive assay. Importantly, there was a significant group of PCR-positive dogs that were considered seronegative (based on 2 reactive tests), highlighting the limitations of current serological testing. However, there was a stronger agreement between serology and PCR when using a single reactive test to identify seropositive dogs compared to using two tests. Thus, it is likely that many of the dogs seropositive with a single test were not false positives but indeed infected with *T. cruzi.* PCR-positive dogs with negative serology may also correspond to acute cases, although their number is higher than the estimated incidence of new infections (see below). Nonetheless, we also observed a tendency of a higher discordance among serological tests with dog age, possibly indicating seroconversion following recent infection.

Our observations confirm that *T. cru*zi infection is widespread in shelter dogs from southern Louisiana, as all shelters are affected (except two, in Acadia and St. Landry parishes, most likely due to a small sample size, *n* = 8 and *n* = 6, respectively). Accordingly, we did not detect any significant association of *T. cruzi* infections with ecoregions. While we cannot determine if dogs became infected at the shelters or prior to their arrival, prior infections would most likely have occurred within the same parish and ecoregion because shelters only accept dogs from the parish where they are located. Also the prevalence of canine *T. cruzi* infection was very similar to that observed in Texas, in spite of the important difference in triatomine vectors. Indeed, *T. sanguisuga* is the main vector in Louisiana [41], while *T. gerstaeckeri* is more frequent in Texas [42].

*Trypanosoma cruzi* infection increased with dog age, as expected from cumulative exposure. This allowed to precisely estimate a high incidence of new cases reaching 2.27% per year. Male dogs were also more infected than females, which is a somewhat unusual observation as sex differences in infection rates are usually not observed [11, 43, 44], and it is not clear if it reflects greater exposure to vectors and/or greater susceptibility to infection of male dogs.

## Conclusions

We documented a high prevalence of *T. cruzi* infection in shelter dogs in southern Louisiana, USA, with at least 6.9% seropositive and 15.7% PCR-positive animals. Therefore, *T. cruzi* infection appears as a very significant and widespread veterinary problem in dogs in the region, although it is mostly unnoticed and underdiagnosed by health professionals. It is also very likely that such *T. cruzi* infections occur in most of the southern USA where triatomine vectors are present [45]. Based on a total population estimated at nearly 90 million pet dogs the USA, many of those living in the southern states may be infected with *T. cruzi*. This highlights the need for greater awareness among the veterinary community for case detection and care, as well as among dog owners to reduce the risks of infection in regions where *T. cruzi* infection is prevalent. While therapeutic treatment may be of limited efficacy [46], insecticide-treated collars may help reduce canine exposure to triatomines and subsequent infection [47, 48]. Alternatively, the development of a veterinary vaccine may help protect dogs from *T. cruzi* infection and disease progression [49, 50].

## Additional file

**Additional file 1: Table S1.** Individual serological and PCR testing for *T. cruzi* in dogs.

## Data Availability

Data supporting the conclusions of this article are included within the article and its additional file. The datasets used and/or analysed during the present study are available from the corresponding author on reasonable request.

## Abbreviations

ELISA: enzyme-linked immunosorbent assay
CI: confidence interval
PCR: polymerase chain reaction
WB: Western blot

## References

[1] WHO (2010). Chagas disease in Latin America: an epidemiological update based on 2010 estimates. Wkly Epidemiol Rec..

[2] Garcia MN, Aguilar D, Gorchakov R, Rossmann SN, Montgomery SP, Rivera H (2015). Evidence of autochthonous Chagas disease in southeastern Texas. Am J Trop Med Hyg..

[3] Montgomery SP, Parise ME, Dotson EM, Bialek SR (2016). What do we know about Chagas disease in the United States?. Am J Trop Med Hyg..

[4] Mott KE, Mota EA, Sherlock I, Hoff R, Muniz TM, Oliveira TS (1978). *Trypanosoma cruzi* infection in dogs and cats and household seroreactivity to *T. cruzi* in a rural community in northeast Brazil. Am J Trop Med Hyg..

[5] Tenney TD, Curtis-Robles R, Snowden KF, Hamer SA (2014). Shelter dogs as sentinels for *Trypanosoma cruzi* transmission across Texas. Emerg Infect Dis..

[6] Gurtler RE, Cecere MC, Lauricella MA, Cardinal MV, Kitron U, Cohen JE (2007). Domestic dogs and cats as sources of *Trypanosoma cruzi* infection in rural northwestern Argentina. Parasitology..

[7] Burkholder JE, Allison TC, Kelly VP (1980). *Trypanosoma cruzi* (Chagas) (Protozoa: Kinetoplastida) in invertebrate, reservoir, and human hosts of the lower Rio Grande valley of Texas. J Parasitol..

[8] Williams GD, Adams LG, Yaeger RG, McGrath RK, Read WK, Bilderback WR (1977). Naturally occurring trypanosomiasis (Chagasʼ disease) in dogs. J Am Vet Med Assoc..

[9] Beard CB, Pye G, Steurer FJ, Rodriguez R, Campman R, Peterson AT (2003). Chagas disease in a domestic transmission cycle, southern Texas, USA. Emerg Infect Dis..

[10] Meyers AC, Meinders M, Hamer SA (2017). Widespread *Trypanosoma cruzi* infection in government working dogs along the Texas-Mexico border: discordant serology, parasite genotyping and associated vectors. PLoS Negl Trop Dis..

[11] Curtis-Robles R, Zecca IB, Roman-Cruz V, Carbajal ES, Auckland LD, Flores I (2017). *Trypanosoma cruzi* (agent of Chagas disease) in sympatric human and dog populations in ‟Colonias” of the lower Rio Grande valley of Texas. Am J Trop Med Hyg..

[12] Curtis-Robles R, Snowden KF, Dominguez B, Dinges L, Rodgers S, Mays G (2017). Epidemiology and molecular typing of *Trypanosoma cruzi* in naturally-infected hound dogs and associated triatomine vectors in Texas, USA. PLoS Negl Trop Dis..

[13] Kjos SA, Snowden KF, Craig TM, Lewis B, Ronald N, Olson JK (2008). Distribution and characterization of canine Chagas disease in Texas. Vet Parasitol..

[14] Meurs KM, Anthony MA, Slater M, Miller MW (1998). Chronic *Trypanosoma cruzi* infection in dogs: 11 cases (1987–1996). J Am Vet Med Assoc..

[15] Barr SC (2009). Canine Chagasʼ disease (American trypanosomiasis) in North America. Vet Clin North Am Small Anim Pract..

[16] Ikenga JO, Richerson JV (1984). *Trypanosoma cruzi* (Chagas) (Protozoa: Kinetoplastida: Trypanosomatidae) in invertebrate and vertebrate hosts from Brewster county in Trans-Pecos Texas. J Econ Entomol..

[17] Kjos SA, Marcet PL, Yabsley MJ, Kitron U, Snowden KF, Logan KS (2013). Identification of bloodmeal sources and *Trypanosoma cruzi* infection in triatomine bugs (Hemiptera: Reduviidae) from residential settings in Texas, the United States. J Med Entomol..

[18] Gorchakov R, Trosclair LP, Wozniak EJ, Feria PT, Garcia MN, Gunter SM (2016). *Trypanosoma cruzi* infection prevalence and bloodmeal analysis in triatomine vectors of Chagas disease from rural peridomestic locations in Texas, 2013–2014. J Med Entomol..

[19] Hodo CL, Hamer SA (2017). Toward an ecological framework for assessing reservoirs of vector-borne pathogens: wildlife reservoirs of *Trypanosoma cruzi* across the southern United States. ILAR J..

[20] Gates M, Gerhold RW, Wilkes RP, Gulsby WD, Maestas L, Rosypal A (2014). Parasitology, virology, and serology of free-ranging coyotes (*Canis latrans*) from central Georgia, USA. J Wildl Dis..

[21] Yabsley MJ, Noblet GP (2002). Seroprevalence of *Trypanosoma cruzi* in raccoons from South Carolina and Georgia. J Wildl Dis..

[22] Yabsley MJ, Brown EL, Roellig DM (2009). Evaluation of the Chagas Stat-Pak assay for detection of *Trypanosoma cruzi* antibodies in wildlife reservoirs. J Parasitol..

[23] Snider TG, Yaeger RG, Dellucky J (1980). Myocarditis caused by *Trypanosoma cruzi* in a native Louisiana dog. J Am Vet Med Assoc..

[24] Vakalis N, Miller JH, Lauritsen E, Hansen D (1983). Anti-*Trypanosoma cruzi* antibodies among domestic dogs in New Orleans. J Louisiana State Med Soc..

[25] Barr SC, Dennis VA, Klei TR (1991). Serologic and blood culture survey of *Trypanosoma cruzi* infection in four canine populations of southern Louisiana. Am J Vet Res..

[26] Nieto PD, Boughton R, Dorn PL, Steurer F, Raychaudhuri S, Esfandiari J (2009). Comparison of two immunochromatographic assays and the indirect immunofluorescence antibody test for diagnosis of *Trypanosoma cruzi* infection in dogs in south central Louisiana. Vet Parasitol..

[27] Bradley KK, Bergman DK, Woods JP, Crutcher JM, Kirchhoff LV (2000). Prevalence of American trypanosomiasis (Chagas disease) among dogs in Oklahoma. J Am Vet Med Assoc..

[28] Barr SC, Van Beek O, Carlisle-Nowak MS, Lopez JW, Kirchhoff LV, Allison N (1995). *Trypanosoma cruzi* infection in Walker hounds from Virginia. Am J Vet Res..

[29] Patel JM, Rosypal AC, Zimmerman KL, Monroe WE, Sriranganathan N, Zajac AM (2012). Isolation, mouse pathogenicity, and genotyping of *Trypanosoma cruzi* from an English Cocker Spaniel from Virginia, USA. Vet Parasitol..

[30] Rosypal AC, Hill R, Lewis S, Braxton K, Zajac AM, Lindsay DS (2010). *Toxoplasma gondii* and *Trypanosoma cruzi* antibodies in dogs from Virginia. Zoonoses Public Health..

[31] Cardinal MV, Reithinger R, Gurtler RE (2006). Use of an immunochromatographic dipstick test for rapid detection of *Trypanosoma cruzi* in sera from animal reservoir hosts. J Clin Microbiol..

[32] Cruz-Chan JV, Bolio-González ME, Colin-Flores R, Ramirez-Sierra MJ, Quijano-Hernandez IA, Dumonteil E (2009). Immunopathology of natural *Trypanosoma cruzi* infection in dogs. Vet Parasitol..

[33] Moser DR, Kirchhoff LV, Donelson JE (1989). Detection of *Trypanosoma cruzi* by DNA amplification using the polymerase chain reaction. J Clin Microbiol..

[34] Herrera CP, Licon MH, Nation CS, Jameson SB, Wesson DM (2015). Genotype diversity of *Trypanosoma cruzi* in small rodents and *Triatoma sanguisuga* from a rural area in New Orleans, Louisiana. Parasit Vectors..

[35] Enriquez GF, Cardinal MV, Orozco MM, Schijman AG, Gurtler RE (2013). Detection of *Trypanosoma cruzi* infection in naturally infected dogs and cats using serological, parasitological and molecular methods. Acta Trop..

[36] Lauricella MA, Castanera MB, Gurtler RE, Segura EL (1998). Immunodiagnosis of *Trypanosoma cruzi* (Chagasʼ disease) infection in naturally infected dogs. Mem Inst Oswaldo Cruz..

[37] Dumonteil E, Herrera C (2017). Ten years of Chagas disease research: looking back to achievements, looking ahead to challenges. PLoS Negl Trop Dis..

[38] Pronovost H, Peterson AC, Ghersi Chavez B, Blum MJ, Dumonteil E, Herrera C (2019). Deep sequencing reveals multiclonality and new discrete typing units of *Trypanosoma cruzi* in rodents from the southern United States. J Microbiol Immunol Infect..

[39] Herrera C, Majeau A, Didier P, Falkenstein KP, Dumonteil E (2019). *Trypanosoma cruzi* diversity in naturally infected non-human primates in Louisiana assessed by deep sequencing of the mini-exon gene. Trans R Soc Trop Med Hyg..

[40] Majeau A, Herrera C, Dumonteil E (2019). An improved approach to *Trypanosoma cruzi* molecular genotyping by next-generation sequencing of the mini-exon gene. Methods Mol Biol..

[41] Cesa K, Caillouet KA, Dorn PL, Wesson DM (2011). High *Trypanosoma cruzi* (Kinetoplastida: Trypanosomatidae) prevalence in *Triatoma sanguisuga* (Hemiptera: Redviidae) in southeastern Louisiana. J Med Entomol..

[42] Curtis-Robles R, Auckland LD, Snowden KF, Hamer GL, Hamer SA (2018). Analysis of over 1500 triatomine vectors from across the US, predominantly Texas, for *Trypanosoma cruzi* infection and discrete typing units. Infect Genet Evol..

[43] Pineda V, Saldana A, Monfante I, Santamaria A, Gottdenker NL, Yabsley MJ (2011). Prevalence of trypanosome infections in dogs from Chagas disease endemic regions in Panama, Central America. Vet Parasitol..

[44] Berrizbeitia M, Concepcion JL, Carzola V, Rodriguez J, Caceres A, Quinones W (2013). Seroprevalencia de la infección por *Trypanosoma cruzi* en *Canis familiaris* del estado Sucre, Venezuela. Biomedica..

[45] Bern C, Kjos S, Yabsley MJ, Montgomery SP (2011). *Trypanosoma cruzi* and Chagasʼ disease in the United States. Clin Microbiol Rev..

[46] Santos FM, Mazzeti AL, Caldas S, Goncalves KR, Lima WG, Torres RM (2016). Chagas cardiomyopathy: the potential effect of benznidazole treatment on diastolic dysfunction and cardiac damage in dogs chronically infected with *Trypanosoma cruzi*. Acta Trop..

[47] Reithinger R, Ceballos L, Stariolo R, Davies CR, Gurtler RE (2005). Chagas disease control: deltamethrin-treated collars reduce *Triatoma infestans* feeding success on dogs. Trans R Soc Trop Med Hyg..

[48] Reithinger R, Ceballos L, Stariolo R, Davies CR, Gurtler RE (2006). Extinction of experimental *Triatoma infestans* populations following continuous exposure to dogs wearing deltamethrin-treated collars. Am J Trop Med Hyg..

[49] Quijano-Hernandez IA, Castro-Barcena A, Vazquez-Chagoyan JC, Bolio-Gonzalez ME, Ortega-Lopez J, Dumonteil E (2013). Preventive and therapeutic DNA vaccination partially protect dogs against an infectious challenge with *Trypanosoma cruzi*. Vaccine..

[50] Dumonteil E, Bottazzi ME, Zhan B, Heffernan MJ, Jones K, Valenzuela JG (2012). Accelerating the development of a therapeutic vaccine for human chagas disease: rationale and prospects. Exp Rev Vacc.

