# Supplementary material for: High prevalence of Trypanosoma cruzi infection in shelter dogs from southern Louisiana, USA
Source: Parasit Vectors. 2019 Jun 25;12:322. doi: 10.1186/s13071-019-3572-y (PMC6593594; doi:10.1186/s13071-019-3572-y)
Supplement: Supplementary file 1 — Additional file 1: Table S1. Individual serological and PCR testing for T. cruzi in dogs. [file 13071_2019_3572_MOESM1_ESM.docx]

**Additional file 1: Table S1. Individual serological and PCR testing for *T. cruzi* in dogs**

| Stat-Pak | ELISA | Western blot | PCR | N |
| --- | --- | --- | --- | --- |
| Neg | Neg | Neg | Neg | 27 |
| Neg | Neg | ND | Neg | 338 |
| Neg | Neg | R | Neg | 31 |
| R | Neg | Neg | Neg | 3 |
| R | Neg | ND | Neg | 15 |
| R | R | Neg | Neg | 1 |
| R | Neg | R | Neg | 6 |
| Neg | R | Neg | Neg | 9 |
| Neg | R | R | Neg | 19 |
| Neg | ND | ND | Neg | 1 |
| R | R | R | Neg | 5 |
| Neg | ND | ND | Pos | 2 |
| Neg | Neg | ND | Pos | 19 |
| Neg | Neg | Neg | Pos | 18 |
| R | Neg | Neg | Pos | 1 |
| Neg | Neg | R | Pos | 35 |
| Neg | R | Neg | Pos | 4 |
| Neg | R | R | Pos | 6 |
|  |  |  |  | **540** |

ND: not done, Neg: negative, R: Reactive, Pos: Positive.
